# From antimicrobial to anticancer peptides. A review

**DOI:** 10.3389/fmicb.2013.00294

**Published:** 2013-10-01

**Authors:** Diana Gaspar, A. Salomé Veiga, Miguel A. R. B. Castanho

**Affiliations:** Instituto de Medicina Molecular, Faculdade de Medicina da Universidade de LisboaLisbon, Portugal

**Keywords:** anticancer peptides, tumor selectivity, electrostatic interactions, membrane disruption, apoptosis induction, necrosis, drug development

## Abstract

Antimicrobial peptides (AMPs) are part of the innate immune defense mechanism of many organisms. Although AMPs have been essentially studied and developed as potential alternatives for fighting infectious diseases, their use as anticancer peptides (ACPs) in cancer therapy either alone or in combination with other conventional drugs has been regarded as a therapeutic strategy to explore. As human cancer remains a cause of high morbidity and mortality worldwide, an urgent need of new, selective, and more efficient drugs is evident. Even though ACPs are expected to be selective toward tumor cells without impairing the normal body physiological functions, the development of a selective ACP has been a challenge. It is not yet possible to predict antitumor activity based on ACPs structures. ACPs are unique molecules when compared to the actual chemotherapeutic arsenal available for cancer treatment and display a variety of modes of action which in some types of cancer seem to co-exist. Regardless the debate surrounding the definition of structure-activity relationships for ACPs, great effort has been invested in ACP design and the challenge of improving effective killing of tumor cells remains. As detailed studies on ACPs mechanisms of action are crucial for optimizing drug development, in this review we provide an overview of the literature concerning peptides' structure, modes of action, selectivity, and efficacy and also summarize some of the many ACPs studied and/or developed for targeting different solid and hematologic malignancies with special emphasis on the first group. Strategies described for drug development and for increasing peptide selectivity toward specific cells while reducing toxicity are also discussed.

## Introduction: leading antimicrobial peptides into anticancer therapy

Inappropriate and irrational use of antibiotics has induced the worldwide emergence and spreading of resistant microorganisms. Nowadays, understanding the biological and biomedical importance of antimicrobial peptides might be regarded as an advance toward new and resistance-free therapies for infectious diseases. Antimicrobial peptides constitute a mechanism of immune defense with low antigenicity (Iwasaki et al., 2009) that can be found in innumerous eukaryotic organisms of different species (Reddy et al., 2004). These are small and generally amphipathic molecules, most of them containing cationic and hydrophobic residues in elevated proportion, thus capable of interacting with microbial membranes (Brandenburg et al., 2012; Seo et al., 2012) through non-specific interactions with the membrane lipids (Arouri et al., 2009). The short time-frame of interaction promotes the microbe rapid death and decreases the probability of resistance development (Fernebro, 2011). There is immense structural diversity in the several hundred AMPs that have been studied until today (Maroti et al., 2011). There are α-helical (such as cecropins), cysteine-rich and β-sheet AMPs (such as defensins). It is also common to find AMPs rich in His, Arg, Pro, and Trp (like indolicidin for instance) (Reddy et al., 2004).

Although Gram-positive and Gram-negative bacteria are the most studied targets for AMPs, many other different targets have been described, like fungi, protozoa (Giuliani et al., 2007) and enveloped viruses, such as HIV and herpes virus (Hancock and Diamond, 2000). The scientific literature is also rich in studies providing information on the mechanisms of action of AMPs (Friedrich et al., 2000; Mika et al., 2011). It is well established that the ability of these small cationic molecules in disrupting and permeating cell membranes is dependent on several biophysical properties, such as peptides' secondary structure, overall net charge, amphipathicity, hydrophobicity, size and balance between hydrophobic and polar regions (Reddy et al., 2004; Teixeira et al., 2012). This ability in permeating the cellular membrane is correlated with the antibiotic effect of several AMPs, such as defensins and cecropins (Steiner et al., 1988; Cociancich et al., 1993). Membrane disruption by AMPs can occur through different modes. It can either consist in pore formation in the lipid membrane (barrel stave and toroidal pore models), thinning of the membrane bilayer, membrane dissolution (carpet model), or lipid-peptide domain formation. In other cases AMPs are capable of intracellular targeting of the pathogen (Yeaman and Yount, 2003; Brogden, 2005; Papo and Shai, 2005; Bechinger and Lohner, 2006; Chan et al., 2006) since AMPs can bind to nucleic acids and proteins (Hancock and Sahl, 2006). There are also reports describing immunomodulatory activities for AMPs (Jerala and Porro, 2004; McPhee et al., 2005) such as the stimulation of chemokine and cytokines production as well as chemotaxis for leukocytes (Bowdish et al., 2005). For further detail on AMPs structures, mechanisms and potential pharmaceutical applications, the reader is referred to the reviews by Li et al. and Brogden et al. (Brogden, 2005; Li et al., 2012). In addition to these well-known and described activities and targets, a growing number of studies report a broad spectrum of cytotoxic activity against cancer cells by these peptides (Moore et al., 1994; Mader and Hoskin, 2006; Hoskin and Ramamoorthy, 2008; Berge et al., 2010).

Cancer remains a major cause of death affecting millions of people and is caused by the growth and spreading of abnormal cells in an uncontrolled manner. Estimates from the international Agency for Research on Cancer (IARC) indicate that 12.7 million of new cancer cases and 7.6 million cancer deaths occurred worldwide during 2008 (Ferlay et al., 2010). Also, the worldwide statistics reveal that the most commonly diagnosed cancers are lung, breast and colorectal (Parkin et al., 2005; Ferlay et al., 2010). In the last decades many effort has been devoted in creating new therapies that are at the same time more selective and less harmful for the patients. Despite this, the methods today available such as surgery and chemotherapy have a relatively low success rate as well as they present a risk of reoccurrence (Harris et al., 2011). Indeed, chemotherapy treatment of prostate, bladder, kidney and pancreatic cancer as well as metastatic melanoma is being inefficient (Riedl et al., 2011a). For these cases where reoccurrence and/or metastasis occur, chemotherapy is the first line of defense (Riedl et al., 2011b). The therapeutic arsenal includes natural products, DNA-alkylating agents, hormone agonists/antagonists and antimetabolites but all of them presenting an insufficient selectivity and consequently an unspecific targeting of healthy mammalian cells with many deleterious effects (Kalyanaraman et al., 2002; Al-Benna et al., 2011). In fact, as chemical agents that are designed to attack the rapidly cancer dividing cells, they are expected to induce side-effects on normal cells that divide at the same rate. Consequently, it is very frequent the occurrence of myelossupression and thrombocytopenia (decreased production of blood cells), mucositis (inflammatory event on the digestive tract) and alopecia (hair loss) due to the non-selective targeting of cells from bone marrow, gastrointestinal tract and hair follicles (Riedl et al., 2011b). Moreover, once many of these compounds pass through the cell membrane and enter the cytosol they are transported back to the outside of the cell as a part of a mechanism of resistance from the cancerous cells (Perez-Tomas, 2006). Besides the increase of the drug transporters that carry the anticancer agent out of the cell, other mechanisms of multiple drug resistance (MDR) may be described. These include the ability of the cell to repair suffered DNA damage, tolerance to stress conditions and abnormal expression of drug detoxifying enzymes (Gatti and Zunino, 2005).

In a time where the number of people suffering from a cancer-related disease increases each day and where conventional therapies gather a worrying number of deficits and drawbacks, new treatment options are a demand for symptoms relieving and ultimately the eradication of the disease. In this context, anticancer peptides have been proved to be a resourceful strategy for the molecularly targeted cancer drug discovery and development process. Small molecules with an efficient tissue penetration and uptake by the heterogeneous cancer cells, endowed with intrinsic activity or synergizing with existing therapeutics, are expected to result in improved anticancer drugs with higher selectivity for neoplastic cells and reduced harmful effects over healthy tissues.

## Anticancer peptides—classification, selectivity, and modes of action

In a structural point of view, most ACPs have either α-helical or β-sheet conformation but some extended structures have already been reported (Hoskin and Ramamoorthy, 2008; Rodrigues et al., 2009; Wang et al., 2009a; Hammami and Fliss, 2010). Concerning cell targets, they can be classified into two major groups. The first one includes peptides active against microbial and cancer cells while not being active against healthy mammalian cells, such as cecropins and magainins. The second group contains ACPs that act against all three types of cells: microbial, normal and cancerous (Papo and Shai, 2005; Hoskin and Ramamoorthy, 2008), such as human neutrophil defensins HNP-1 to 3 (Papo and Shai, 2005; Droin et al., 2009). For a complete list of ACPs the reader is referred to the database available on http://aps.unmc.edu/AP/database/antiC.php.

The mechanism and selectivity criteria by which ACPs kill cancerous cells is still a controversial theme although some major conclusions can be outlined. ACPs oncolytic effects may generally occur either by membranolytic or non-membranolytic mechanisms (Harris et al., 2013). The mechanism underlying each membranolytic peptide activity is dependent on the ACP characteristics as well as on the target membrane features, which in turn modulate peptides' selectivity and toxicity (Schweizer, 2009). In fact, cancer and normal mammalian cells have a number of differences that are accounted responsible for the selectivity of some of the ACPs. These differences rely firstly in the membrane net negative charge that characterizes malignant cells (Schweizer, 2009). Anionic molecules such as the phospholipid phosphatidylserine (PS), O-glycosylated mucins, sialylated gangliosides and heparin sulfate are present in the membrane of cancer cells, conferring them a net negative charge which contrasts with the normal mammalian cell membrane, typically zwitterionic in nature (Hoskin and Ramamoorthy, 2008; Schweizer, 2009). Increased sialic acid content on the membrane affects membrane charge by stimulating surface concentration of acid groups (Dobrzynska et al., 2005). In addition to the modified glycosilation profile typical of cancer tissues and which is directly associated with the tumor phenotype (Dube and Bertozzi, 2005), during cell transformation PS molecules will present themselves on the outer membrane leaflet, accumulating on site and counteracting the typical phospholipid asymmetry of the membrane (Utsugi et al., 1991; Hoskin and Ramamoorthy, 2008; Schweizer, 2009). Along with the zwitterionic lipids, normal cell membranes have high contents of cholesterol which has been proposed as a protective molecule of the membrane by modulating the cell fluidity and blocking the entry/passage of cationic peptides (Schweizer, 2009). On the opposite, most cancer cell membranes are described to be more fluid than normal cells (Kozlowska et al., 1999; Sok et al., 1999) allowing membrane destabilization by ACPs. Nevertheless, there has been also shown that certain tumors, like breast and prostate, present a higher content of cholesterol in the cell membranes (Li et al., 2006) posing an obstacle to the lysis by ACPs. The cell surface area is also a factor controlling ACPs activity since the elevated number and distorted features of microvili present on the malignant cells confer them higher surface area and higher contact with ACPs molecules (Domagala and Koss, 1980; Chaudhary and Munshi, 1995; Chan et al., 1998).

The negative surface charge of the cancer cell membrane is a characteristic also shared by the bacterial cells (Mader and Hoskin, 2006; Hoskin and Ramamoorthy, 2008). This fact lead to the hypothesis that AMPs and ACPs share similar molecular principles for selectivity and activity (van Zoggel et al., 2012). However, not all AMPs are ACPs (Hoskin and Ramamoorthy, 2008) and so it is of crucial importance the comprehension of all factors that allow ACPs to recognize and lyse neoplastic cells for understanding efficacy and selectivity phenomena. Unraveling the specific targets that are expressed and presented within a certain tumor type will be a valuable source of information in the process of drug design.

ACPs' membranolytic and selective mode of action on tumor cells can be due to the increased anionicity of the cytoplasmic membrane of these cells. The same “carpet” and “barrel-stave” models, for instance, used for describing AMPs interaction with bacterial membranes are also applied in this case (Pouny and Shai, 1992; Oren and Shai, 1998; Schweizer, 2009). Further membranolytic events involve the permeation and swelling of mitochondria with release of cytochrome c and apoptosis events (Mai et al., 2001). Although the rapid killing associated to ACPs might imply the prevalence of a non-receptor mediated mode of action, some non-membranolytic activities for ACPs have also been described (Sharma, 1992; Wachinger et al., 1998; Winder et al., 1998). Different attempts in controlling tumorigenesis involve the targeting of the angiogenesis process. Peptides that block the function of receptors expressed on angiogenic endothelial cells and that by this way perturb the formation of the vasculature associated to a tumor have been described (Arap et al., 1998; Mader and Hoskin, 2006; Schweizer, 2009; Lee et al., 2011; Rosca et al., 2011). The main goal nowadays when using an anti-angiogenic therapy is to normalize the tumor vasculature instead of reducing the density of tumor blood vessels (Shang et al., 2012). The development of therapeutic molecules which by their own or in a combination with other chemotherapy agents target several aspects of the angiogenic events might prove fruitful in cancer treatment (Rosca et al., 2011).

## Anticancer peptides for solid and hematological tumors

Regardless the many scientific studies published in which peptides are shown to successfully eliminate tumor cells both *in vitro* and *in vivo* and also prevent metastases formation (Cruciani et al., 1991; Ellerby et al., 2003; Papo et al., 2003, 2004, 2006), there has always been difficulties in establishing a clear structure-activity relationship for ACPs that might facilitate drug development. Targeted peptides which recognize tumors and metastases in a specific manner are difficult to obtain. In this section, information concerning ACPs that have been designed, synthesized or isolated and studied for targeting specific tumor cells is provided. Due to the vast broad spectrum of cancer cells tested for each ACP in study, the different tumors have been divided into two main groups, hematological and solid, and some of the ACPs that have been described to target cells from each group are reviewed with special emphasis on the solid tumors. Selectivity, efficacy and major requirements for anticancer activity are discussed. As the literature is vast concerning this matter, this review is focused in a period covering nearly 20 years of AMP cancer cell treatment. Table 1 shows the primary sequence of some of the peptides with anticancer activity described in this review.

**Table 1:** **Peptides with anticancer activity toward solid and hematological tumors**.

**Peptide name**	**Amino acid sequence**	**References**
D-peptide A	RLYLRIGRR	Iwasaki et al., 2009
D-peptide B	RLRLRIGRR	
D-peptide C	ALYLAIRRR	
D-peptide D	RLLLRIGRR	
D-K6L9	LKLLKKLLKKLLKLL	Papo et al., 2006
NRC-03	GRRKRKWLRRIGKGVKIIGGAALDHL	Hilchie et al., 2011
NRC-07	RWGKWFKKATHVGKHVGKAALTAYL	
Gomesin	ZCRRLCYKQRCVTYCRGR	Rodrigues et al., 2008
Hepcidin TH2-3	QSHLSLCRWCCNCCRSNKGC	Chen et al., 2009
Dermaseptin B2	GLWSKIKEVGKEAAKAAAKAAGKAALGAVSEAV	van Zoggel et al., 2012
PTP7	FLGALFKALSKLL	Kim et al., 2003
MGA2	GIGKFLHSAKKFGKAFVGEIMNSGGKKWKMRRNQF–WVKVQRG	Liu et al., 2013
HNP-1	ACYCRIPACIAGERRYGTCIYQGRLWAFCC	Wang et al., 2009c
Tachyplesin	KWCFRVCYRGICYRRCR	Chen et al., 2005
Temporin-1CEa	FVDLKKIANIINSIF	Wang et al., 2012
NK-2	KILRGVCKKIMRTFLRRISKDILTGKK	Schroder-Borm et al., 2005
Bovine lactoferricin B6 (Lbcin B6)	RRWQWR	Richardson et al., 2009
Cecropin CB1	KWKVFKKIEKMGRNIRNGIVKAGPKWKVFKKIEK	Srisailam et al., 2000

### Solid tumors

Solid tumors are characterized by a mass of tissue without cysts or liquid areas. In these tumors it is possible to distinguish malignant cells and the stroma where these cells are maintained (Dvorak et al., 1986). The tumor masses are represented by phenotypically heterogeneous populations of cancer cells, each having its own ability in proliferating and forming a new tumor (Reya et al., 2001; Al-Hajj and Clarke, 2004). The physiology and morphology of these tumors is largely deviated from the normal tissues (Brown and Giaccia, 1998) and these differences are currently being explored for selective cancer treatments in an attempt of circumvent the low specificity of the actual chemical and radiation therapy. Regarding the use of peptides in cancer therapy, many ACPs have been developed for targeting different types of solid tumors. Conclusions about structure requirements for the selective targeting of this type of tumors remain elusive. Available results show that ACPs target solid tumors by a variety of mechanisms.

Breast and prostate cancers are the most frequently diagnosed cancer in women and men aside from skin cancer (Jemal et al., 2006; Gross and Andra, 2012). Estimates indicate that breast and prostate cancers accounted for 23 and 14% of the total new cancer cases only in 2008 (Ferlay et al., 2010). Moreover, prostate cancer does not respond adequately to single or multiple drug regimens (Papo et al., 2004). Breast, prostate, uterine cervix, liver and lung are some of the targeted tumors for the development of ACPs. Some of these peptides defy the malignant cells by apoptotic or necrotic mechanisms after damaging cellular membranes, others by intracellular targets and it is also possible that one single peptide presents more than one mode of anticancer activity. Table 2 summarizes some of the ACPs studied in the targeting of solid tumors.

**Table 2:** **Peptides and their respective oncolytic properties against solid tumors**.

**Peptide**	**Cancer cell**	**Experimental test**	**Selectivity**	**Anticancer activity**	**References**
D-peptides A, B, C and D	Human cervix, glioma, lung, mouse myeloma, african green monkey kidney	ICL	Yes	Cell membrane disruption	Iwasaki et al., 2009
D-K_6_L_9_	Human prostate	ICL/GEM	Yes	Necrosis via membrane depolarization	Papo et al., 2006
NRC-03, NRC-07	Human breast	ICL/GEM	No	Cell membrane lysis with possible pore formation in mitochondria and ROS production	Hilchie et al., 2011
MPI-1	Human cervix, prostate and hepatocellular adenocarcinoma,	GEM	Yes	Necrosis after cell membrane targeting	Zhang et al., 2010
Polybia-MPI	Human bladder and prostate	ICL	Yes	Cell membrane disruption with probable pore formation	Wang et al., 2008
Gomesin	Murine melanoma, human breast, colon and cervix adenocarcinoma	ICL	Nd	Unclear; possible pore formation	Rodrigues et al., 2008
Hepcidin TH2-3	Human cervix, hepatocellular carcinoma, fibrosarcoma	ICL	Yes	Cell membrane lysis	Chen et al., 2009
SVS-1	Human lung, epidermis and breast	ICL	Yes	Cell membrane disruption via pore formation	Gaspar et al., 2012; Sinthuvanich et al., 2012
Epinecidin-1	Human lung, cervix, hepatocellular carcinoma, fibrosarcoma, histiocytic lymphoma	ICL	Yes	Cell membrane lysis mediated by necrosis inhibitory activity	Lin et al., 2009
Dermaseptin B2	Human prostate and breast	ICL/GEM	Yes	Necrosis	van Zoggel et al., 2012
PTP7	Human lung, prostate, breast and hepatocellular carcinoma	ICL	Yes	Apoptosis induction	Kim et al., 2003
BEPT II and BEPT II-1	Human prostate	ICL	Nd	Apoptosis induction	Ma et al., 2013
TfR-lytic peptide	Human breast and prostate, gliobastoma, pancreas and bile-duct	ICL/GEM	Yes	Apoptosis induction	Kawamoto et al., 2011
BPC96	Human cervix	ICL	Yes	Apoptosis induction	Feliu et al., 2010
RGD-Tachyplesin	Human prostate, melanoma	ICL/GEM	Some selectivity	Apoptosis induction	Chen et al., 2001
MG2A	Human cervix and lung, melanoma, rat glioma	ICL/GEM	Yes	Both necrosis and apoptosis	Liu et al., 2013
A_9_K	Human cervix, kidney	ICL	Yes	Both necrosis and apoptosis	Xu et al., 2013
HNP-1	Mouse colon and breast	GEM	Nd	Mediation of antitumor immunity	Wang et al., 2009c
Hecate, Phor14 and Phor21 -BCG	Human prostate, breast, ovarian and testicular cells	ICL/GEM	Yes	Necrosis	Leuschner et al., 2003; Leuschner and Hansel, 2005; Hansel et al., 2007
Myristoyl-Cys-Ala-Val-Ala-Tyr-(1,3 dimethyl)His-OMe	Several human cell lines (lung, colon, breast, ovarian, renal, …)	ICL	Nd	DNA synthesis/replication inhibition	Ourth, 2011
9 somatostatin peptide analogues	Human colon	ICL	Yes	DNA polymerase β nhibition	Kuriyama et al., 2013
Pentastatin-1, chemokinostatin-1, properdistatin	Human breast	ICL/GEM	Nd	Tumor growth and angiogenesis inhibition	Koskimaki et al., 2009
ERα17p	Human breast	ICL/GEM	Nd	Apoptosis induction and massive necrosis	Pelekanou et al., 2011; Byrne et al., 2012
A-8R	Human prostate	ICL/GEM		ROS generation and DNA damage	Gao et al., 2013
CR1166	Human breast and pancreas	ICL	Yes	Apoptosis induction	Patra et al., 2012
Peptide aptamers	Human cervix, mouse melanoma, rat colon	ICL/GEM	Nd	Apoptosis induction by inhibition of HSP-70	Rerole et al., 2011
Tachyplesin	Human prostate	ICL	Nd	Activation of the classic complement pathway	Chen et al., 2005
Temporin-1CEa	Human breast	ICL	Yes	Membrane disruption, calcium release, ROS production	Wang et al., 2012, 2013a,b

Nd, not determined; ICL, immortal cancer-cell lineage; GEM, grafts experimental model (xenografts).

#### Electrostatic interactions are key activity modulators

In a pioneer study, Iwasaki et al. evaluated by flow cytometry the PS density on the surface of several cancer cell lines and established a correlation with cell sensitivity to AMPs using four enantiomeric AMP analogs derived from beetle defensins (D-peptides A, B, C, and D) (Iwasaki et al., 2009). The results showed a selective cytotoxic activity dependent on the negative charge of the cancer cell membrane and PS, providing direct proof of PS role in the anticancer activity (Iwasaki et al., 2009). Papo et al. had also reported on a short host defense-like peptide that inhibits the growth of primary human prostate and breast carcinomas after being injected intratumorally (Papo et al., 2004, 2006). The D-K_6_L_9_ peptide induced a two-step cytolytic effect via membrane disruption and necrosis of tumor cells alongside with a decrease in the tumor vessel density, formation of new capillary tubes and in the secretion of prostate-specific antigen secretion. It was also observed a decrease in the spontaneous and experimental metastases formation (Papo et al., 2006). PS was identified as the target for this peptide which colocalizes with the negatively charged phospholipid and exerts a membrane-depolarizing lytic activity on the neoplastic cells interacting with them in a selective way (Papo et al., 2006). Further than PS molecules, many other membrane components which contribute to the negative membrane of the malignant cell have been selected as anticancer activity triggers. Binding to negatively charged gangliosides expressed on the cell surface can be a potential alternative for directing ACPs activity and a source of information to elucidate the mechanisms by which AMPs bind to normal and cancerous cells (Hanai et al., 2000; Bitton et al., 2002; Guo and Wang, 2009; Miyazaki et al., 2012). NRC-03 and NRC-07 are two peptides from the AMP pleurocidin family with activity against human breast cancer cells including drug-resistant variants and with decreased affinity toward human healthy cells even by intratumoral administration (Hilchie et al., 2011). Peptides are able to bind the cancer cells and cause membrane effects through negatively-charged molecules that are exposed on the cells' membrane, specifically heparan and chondroitin sulfate. Cell death also involved mitochondrial damage and reactive oxygen species (ROS) production (Hilchie et al., 2011). Many other peptides were described, such as MPI-1 (Zhang et al., 2010), Gomesin (Rodrigues et al., 2008), tilapia hepcidin TH2-3 (Chen et al., 2009) and SVS-1 (Gaspar et al., 2012; Sinthuvanich et al., 2012), that seem to target cancer cells on the basis of charge rather than cell growth. MPI-1 is an analog of the AMP polybia-MPI, a peptide isolated from the venom of the wasp *Polybia paulista* (Wang et al., 2009b). MPI-1 peptide has a thioamide bond substitution that selectively binds to human prostate and liver cancer cells causing injury, swelling, bursting, and final cell death by necrosis (Zhang et al., 2010). Scanning electron microscopy (SEM) studies show the disruption of the cell membrane and the authors point the peptide amphipatic α-helical structure as crucial for its activity as well as the surface charge of the cell (Zhang et al., 2010). It is also shown that the thioamide bond substitution can be a valid strategy for designing ACPs representing a conservative modification of the peptide backbone structure (Zhang et al., 2010). The same authors have previously demonstrated that the original peptide, polybia-MPI selectively inhibited the proliferation of prostate and bladder cancer cells with low cytotoxicity for normal murine fibroblasts and that the α-helical conformation was an important feature for achieving an anticancer effect (Wang et al., 2008). The exposure of PS on the cells' membranes was suggested as a possible trigger for the peptides selective killing ability (Wang et al., 2008).

**Figure 1:**
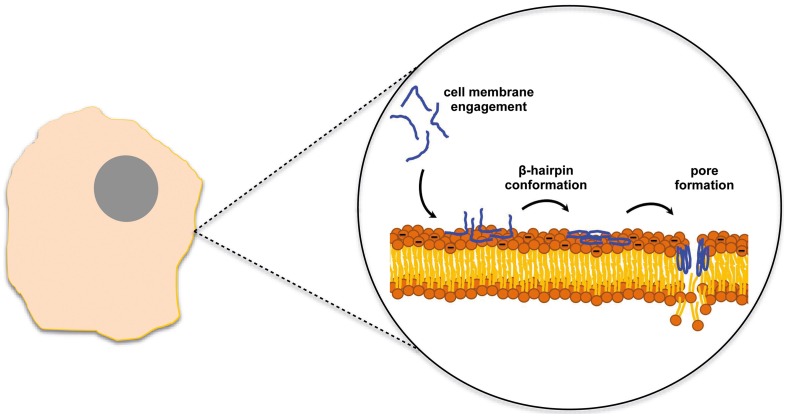
**SVS-1 anticancer peptide disrupts the cell membrane after engaging the membrane surface and folding into a β-hairpin conformation (Sinthuvanich et al., 2012)**.

**Figure 2:**
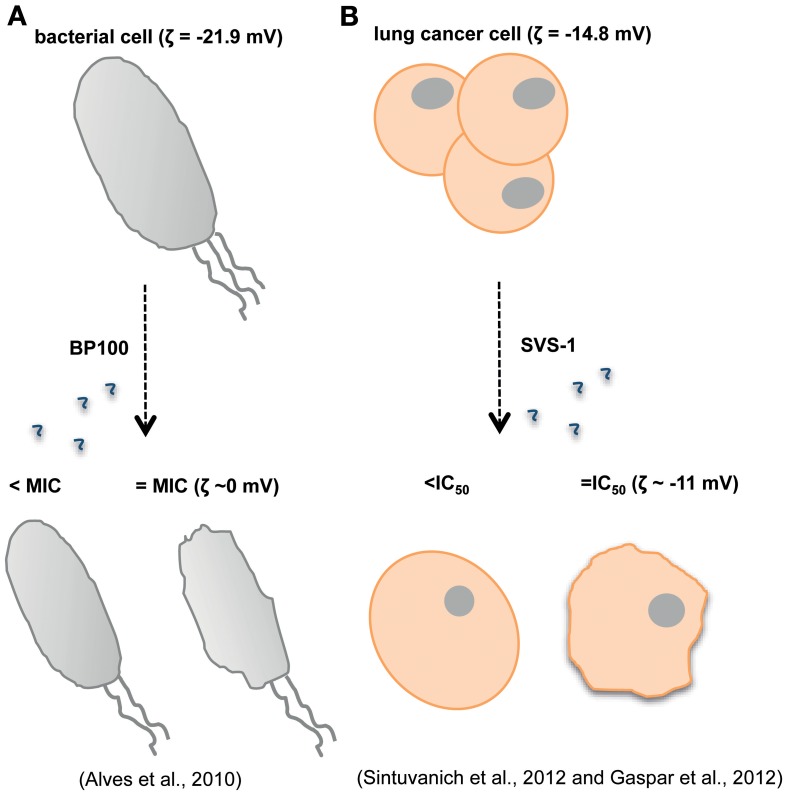
**Antimicrobial and anticancer peptides (AMPs and ACPs) are expected to show different modes of action**. While for the BP100 AMP is possible to establish a correlation between the minimal inhibitory concentration (MIC) and membrane charge (Alves et al., 2010) **(A)**, for the ACP SVS-1 membrane neutralization does not occur even after cell death **(B)** (Gaspar et al., 2012; Sinthuvanich et al., 2012).

Gomesin is a cationic AMP with a hairpin-like two-stranded antiparallel β-sheet structure isolated from hemocytes of *Acanthoscurria gomesiana* (Rodrigues et al., 2008). Rodrigues et al. showed that this AMP possesses anticancer activity *in vivo* after topical treatment for subcutaneous murine melanoma and *in vitro* for melanoma, breast and colon carcinomas (Rodrigues et al., 2008). Although the precise mode of action is not described and may include pore formation, the cytotoxic activity was dependent on the β-hairpin structure and electrostatic forces as well as hydrophobic interactions which were already proved to be important factors for the AMP activity (Fazio et al., 2007). Most of the AMPs active against tumor cells adopt either a bioactive helical conformation at the cell surface or a β-sheet structure prior to engaging the membrane. SVS-1 peptide, a small designed anticancer peptide, folds only at the surface of cancer cells and acquires a β-sheet structure that disrupts the cell membrane via pore formation (Figure 1) (Sinthuvanich et al., 2012). The published studies show that this small 18-residue ACP folding is electrostatically induced and cell death occurs before the peptide neutralizes the cells' negative membrane charge (Gaspar et al., 2012). SVS-1 showed cytotoxic activity against lung, epidermal and breast carcinoma cells and low toxicity against healthy cells (HUVEC and erythrocytes) (Gaspar et al., 2012; Sinthuvanich et al., 2012). Recent studies point to a neutralization of the bacterial membrane charge that coincides or is closely related to minimal inhibitory concentration (MIC) values (Alves et al., 2010) contrary to what might happen with ACPs (Figure 2). Therefore, SVS-1 studies together with others which have been conducted with different types of peptides (Kim et al., 2003) clearly show that antitumor cell activities may actually not parallel AMPs mode of action and that differences should be expected. Furthermore, the different expression patterns of negatively charged molecules on cancer cell membranes will be a limiting factor conditioning the binding and engagement of peptides in the membrane and consequently dictating the ability of each peptide in killing specific cells. This points to the possibility of the same peptide to act by different mechanisms depending on the cell type in question (Yoo et al., 1997; Eliassen et al., 2006) and to be selective against determined types of cancer.

#### Interfering with necrosis and apoptosis mechanisms

After engagement of ACPs in the cell membrane, the peptides may penetrate into intracellular spaces. This entrance might lead to the disruption of the cell membrane accompanied by pore formation and/or changes on the cell membrane charge (Janek et al., 2013) and finally the interference with necrotic (Maher and McClean, 2008; Huang et al., 2011; Ausbacher et al., 2012; Wang et al., 2013a) and apoptotic pathways (Ausbacher et al., 2012). Pore formation is a mechanism associated to some AMPs and has been reported for ACPs as well (Rodrigues et al., 2009). The insertion of bulky hydrophobic amino acids on the cell membrane hydrophobic core with the acquisition of a stable structure can be the driving events for pore formation (Hilchie et al., 2011). Cell death might be a result of apoptosis and/or necrosis, which are characterized by different cellular morphological changes (Elmore, 2007). Indeed, apoptosis of cancer cells is a recognized strategy in cancer therapy (Qiao and Wong, 2009). While analyzing the effects that ACPs exert upon cancer and healthy cells using microscopy and fluorescence tools, it is common to search for cell shrinkage or swelling, chromatin condensation, cytoplasmic vacuoles or even membrane blebbing (Elmore, 2007). There are many peptides capable of inducing these cellular changes. For instance, the synthesized AMP epinecidin-1 selectively kills cancer cells at low concentration and studies of necrosis inhibition test and real-time PCR indicate a membrane disruptive activity as well as an anti-necrosis effect by inhibition of necrosis gene expression (Lin et al., 2009). Dermaseptin B2 is one other example of a necrosis-inducing peptide. Increased lactate dehydrogenase (LDH) release, the positive staining with propidium iodide (PI) as well as confocal microscopy studies points to a necrotic mechanism which in turn might be induced after binding and disruption of the plasma membrane (van Zoggel et al., 2012). Also, induced apoptosis in several human cancer cell lines from breast, uterine cervix, liver and prostate has been described as the mode of action for different potential ACPs (Kim et al., 2003; Feliu et al., 2010; Kawamoto et al., 2011; Ma et al., 2013). It is also possible to attack metastatic tumor cells with peptide-induced apoptosis (Yang et al., 2008) as well as induce apoptosis in the tumor associated endothelial cells (Chen et al., 2001). As metastases are responsible for increased therapy failure, peptides that can specifically intervene in the process of metastases formation stimulating neoplastic cell death are valuable resources in cancer treatment. Chen et al. studied the effect of RGD-tachyplesin on human prostate cancer and melanoma cells (Chen et al., 2001). In this study, the natural tachyplesin was linked to a homing domain which facilitates the AMP internalization into the cells by the binding to integrins on tumor and endothelial cells (RGD sequence). Results showed that the peptide inhibited not only the growth of cancer cells both *in vivo* and *in vitro* with some degree of selectivity, but also affected membrane function triggering apoptosis (Chen et al., 2001).

Peptides that share both necrotic and apoptotic modes of action have been also described. The conjugation of magainin II (MG2) with the N-terminus of the cell penetrating peptide penetratin (Antp) resulted in a fusion peptide, MG2A, active against tumor cells with an IC_50_ in the micromolar range that target chondroitin sulfate on the surface of tumor cells (Liu et al., 2013). Tests involving apoptosis assessment by annexin V and PI staining, fluorescence microscopy and FACS analysis suggest necrotic cell death by membrane lysis while observing apoptotic cells. A different peptide also with dual mode of action was described by Xu et al. (2013). A_9_K is a short designed amphiphilic AMP which combines a short length with other properties such as inherent surfactant-like and AMP activities, protease stability and absence of immunological responses. Presents high selectivity for leukemia, uterine cervix and kidney cancer cells killing cells by membrane disruption and apoptosis (Xu et al., 2013).

#### Diversified modes of action and molecular targets

**Figure 3:**
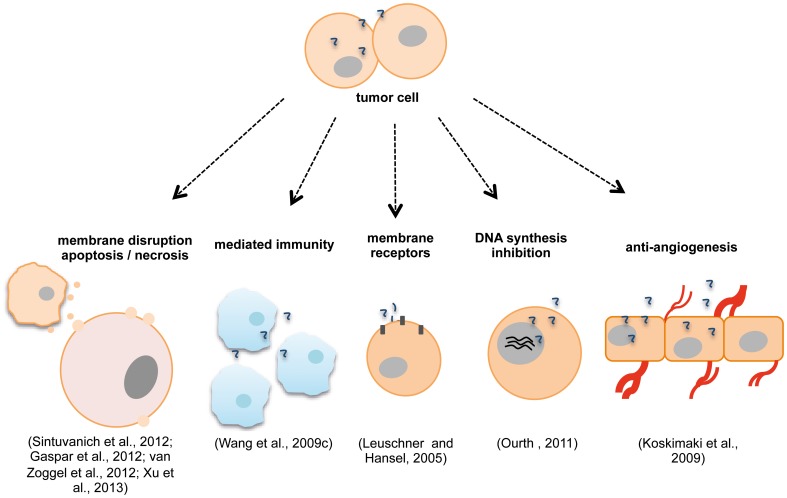
**Anticancer peptides (ACPs) modes of action may include disruption of plasma/ mitochondrial membranes (Sinthuvanich et al., 2012), necrosis, apoptosis (van Zoggel et al., 2012; Xu et al., 2013), mechanisms of mediated immunity (Wang et al., 2009c), membrane receptors involvement (Leuschner and Hansel, 2005), inhibition of DNA synthesis (Ourth, 2011) and anti-angiogenic effects (Koskimaki et al., 2009)**. Different ACPs can act by more than one mechanism simultaneously (Wang et al., 2009c; Xu et al., 2013).

The modes of action for ACPs are not limited only to the disruption of the plasma and mitochondrial membranes with the subsequent damages above-mentioned. Other mechanisms do exist and have been described, while it is not unusual to find peptides that combine more than one mechanism. These may involve alternative pathways such as, mediated immunity (Wang et al., 2009c), hormonal receptors (Leuschner and Hansel, 2005), DNA synthesis inhibition (Ourth, 2011; Kuriyama et al., 2013) and anti-angiogenic effects (Koskimaki et al., 2009) (Figure 3). Indeed, one study showed that human neutrophil peptides HNP-1 to 3 can exert several antitumor effects and that these might occur by a variety of mechanisms (Wang et al., 2009c). HNP-1 to 3 belong to the α-defensin group and are potent AMP with ~30 amino acid residues (Droin et al., 2009). Different studies revealed that these peptides have potential as cancer prognostic markers (Albrethsen et al., 2005, 2006; Droin et al., 2009), are active against a variety of healthy and malignant mammalian cells (Nishimura et al., 2004; McKeown et al., 2006) and that they are found in the tissue of epithelial tumors as well as they are associated with tumor necrosis when expressed intratumorally (Bateman et al., 1992; Muller et al., 2002). In this particular study, Wang et al. showed that the expression of mature HNP-1 in models of breast and colon tumors induced the recruitment and activation of dendritic cells which led to an immune response to the tumor from the host. HNP-1 intratumoral expression in its mature form may inhibit and eradicate established tumors (Wang et al., 2009c). Increased apoptosis and decreased angiogenesis events are also reported with the antitumor effects. Other studies reveal the potential in using peptides that target or mimic hormonal receptors and hormonal-regulated genes for treating cancer (Leuschner et al., 2003; Leuschner and Hansel, 2005; Hansel et al., 2007; Kampa et al., 2011; Pelekanou et al., 2011; Byrne et al., 2012; Gao et al., 2013). Leuschner et al. studied the ability of a series of compounds formed by synthetic membrane-disrupting peptides and a 15-amino acid residues segment of the beta chain of chorionic gonadotropin in targeting cells expressing luteinizing hormone/chorionic gonadotropin (LH/CG) receptors (Leuschner and Hansel, 2005; Hansel et al., 2007). These formed conjugates were able to destroy metastases and disseminated cells derived from human prostate cancer xenografts in nude mice and cells died by necrosis as revealed by histological examinations (Leuschner and Hansel, 2005). These studies prove that the lytic peptide conjugates might be useful for the inhibition of the development of metastases after surgical removal of the primary tumor (Hansel et al., 2007). In a different study, the ERα17p peptide originated from part of the sequence of the estrogen receptor α (ERα) was shown to interact with the polar part of the plasma cell membrane, to penetrate it and induce cell membrane damage at high concentration (Byrne et al., 2012).

Many other targets can be found for developing specific ACPs and many proteins have been highlighted for this effect. Patra et al. reported on the cell-permeable lipopeptide CR1166 that inhibits the PDZ domain of the GIPC (GAIP-interacting protein, C terminus) protein which is over expressed in breast and pancreas tumors (Patra et al., 2012). This inhibition interferes with the protein-protein interactions disturbing the events involved with GIPC activity and which in turn include tumor progression (Patra et al., 2012). Effects were observed *in vivo* and *in vitro* and account for decreased proliferation, cytotoxic effects and apoptosis on breast and pancreas cancer cells (Patra et al., 2012). The heat shock protein 70 (HSP70) has been also described as a potential protein target for treating neoplastic diseases (Rerole et al., 2011), since its upregulation induces an increased tumorigenicity of cancer cells in rodent models (Jaattela, 1995) and its downregulation kills cancer cells or renders them susceptible to apoptosis (Nylandsted et al., 2000). Molecules that inhibit matrix metalloproteinases (MMPs) activity are also interesting potential drugs (Destouches et al., 2012). MMPs are a family of membrane-bound zinc endopeptidases that display an important activity in what concerns remodeling of the extracellular matrix (ECM) in processes as tumor development, angiogenesis and metastatic progression (Coussens and Werb, 2002; Coussens et al., 2002; Egeblad and Werb, 2002; Visse and Nagase, 2003; Jang et al., 2011; Destouches et al., 2012). Many cancers express aberrant MMPs quantities (Derrico et al., 1991; Davies et al., 1993) and this fact can be used for creating strategies to block metastasis process. For instance, buforin IIb, that displays activity against 60 human tumor cell lines (Lee et al., 2008), was fused with a modified magainin sequence, a negative charge that would equilibrate the overall positive charge of buforin II, generating the MMIS:buforin IIb fusion peptide. Both peptide sequences were in turn linked by an octapeptide, cleavable by the MMP-2 (gelatinase A) and MMP-9 (gelatinase B) enzymes which are over expressed in tumor tissues, allowing the release of buforin IIb (Jang et al., 2011). Cells expressing high amounts of MMPs such as mouse melanoma, human fibrosarcoma and gliobastoma were sensitive to this peptide. On the other hand, human uterine cervix cells which deficiently express these MMPs were resistant, and the fusion peptide anticancer activity was shown to be dependent on enzymatic activity (Jang et al., 2011). Two final different examples concerns to tachyplesin and temporin-1CEa. Tachyplesin is a peptide with 17 amino acid residues isolated from the horseshoe crab, which revealed an antitumor activity connected to a binding to hyaluronan or related glycosaminoglicans on the surface of cells and activation of the classic complement pathway leading to the disruption of the plasma membrane (Chen et al., 2005). The AMP temporin-1CEa causes breast cancer cell death by significant membrane disruption, intracellular calcium release and ROS over production (Wang et al., 2012, 2013a,b).

### Hematological tumors: leukemias, myelomas and lymphomas

Hematological malignancies consist in a broad spectrum of diseases which comprise blood, bone marrow and lymph nodes cancer and are classified as leukemia, myeloma and lymphoma, respectively (Alvarez-Calderon et al., 2013). Estimates indicate a 3.4% of deaths caused by leukemia in 2008 (Ferlay et al., 2010). At the present, hematologic cancer is treated with cytotoxic drugs, radiation therapy or with bone marrow transplantation, which is known to cause severe long-term effects on patients (Alvarez-Calderon et al., 2013). As a complex group of diseases affecting multiple cell types, the literature provides numerous examples of peptides developed to target blood and bone marrow cells, many of them designed also to be active against solid tumors (Table 3).

**Table 3:** **Peptides and their respective oncolytic properties against hematological tumors**.

**Peptide**	**Cancer cell**	**Experimental test**	**Selectivity**	**Anticancer activity**	**References**
NK-2	Human chronic myelogenous leukemia, histiocytic lymphoma, acute T cell leukemia, acute lymphoblastic leukemia, neuroblastoma, colorectal adenocarcinoma	ICL	Yes	Necrotic death after peptide intercalation into PS-containing membranes	Schroder-Borm et al., 2005
Polycationic peptides	Human acute T cell leukemia	ICL	Yes	Plasma membrane permeabilization by pore formation	Lemeshko, 2013
Polybia-MPI	Human chronic myelogenous leukemia, promyelocytic leukemia, mouse lymphocytic leukemia	ICL	Yes	Disruption of the plasma membrane by pore formation	Wang et al., 2009b
Bovine Lactoferricin (LfcinB)	Human acute lymphoblastic T leukemia, acute T cell leukemia	ICL	Yes	Apoptosis by direct disruption of the mitochondrial membrane	Furlong et al., 2008
Bovine Lactoferricin B6 (LfcinB6)	Human acute T cell leukemia, acute lymphoblastic T leukemia	ICL	Yes	Intracellular cytotoxicity by cathepsin B and caspase activation	Richardson et al., 2009
Cecropin CB1a	Human acute lymphoblastic T-leukemia cells, lung carcinoma, stomach carcinoma	ICL	Yes	Unclear mode of action	Wu et al., 2009
SK84	Human leukemia, liver and breast	ICL		Membrane disruption	Lu and Chen, 2010
Magainin analogues	Human acute T and B cell leukemia, human chronic myelogenous leukemia, human histiocytic/Burkitt lymphoma, Ape T cell leukemia, human breast, prostate and neuroepithelioma	ICL	Yes	Membrane Lysis	Cruciani et al., 1991
Cecropin CB1	Human chronic myelogenous leukemia, acute T cell leukemia, acute lymphoblastic T-leukemia	ICL	Nd	Membrane Lysis	Srisailam et al., 2000
Pep 2 and Pep3	Human chronic myelogenous leukemia, acute lymphoblastic T-leukemia cells	ICL	Yes	Apoptosis of cancer cells through activation of caspases -3 and -9	Edison et al., 2012
BIM SAHB_A_	Human histiocytic lymphoma, chronic myelogenous leukemia, acute myeloid leukemia	CT	Yes	Apoptotic resistance overcoming	Labelle et al., 2012

Nd, not determined; ICL, immortal cancer-cell lineage; CT, clinical trial.

#### The multiple roles of the negatively charged cancer cell membrane

As above-mentioned, the existence of negatively charged molecules on the cancer cell membrane might render cells susceptible to ACPs. Many peptides targeting non-solid tumors take advantage from electrostatic attraction, such as the NK-2 peptide derived from the cationic core region of NK-lysin from porcine and T-cells. NK-2 has a positive net charge and selectively kills cancer cells by a necrotic mechanism (Schroder-Borm et al., 2005). This killing ability correlates with the exposure of negatively charged PS on the surface of the cancer cell and the intercalation of the peptide into PS-containing membranes, being the leukemia cells with lower PS exposure the least sensitive (Schroder-Borm et al., 2005). This study involved also cells from solid tumors and Schroder-Borm et al. equally demonstrated the importance of PS presence on the cell membrane for NK-2 activity toward neuroblastoma cells (Schroder-Borm et al., 2005). The permeabilization of the plasma membrane due to pore formation by peptides was also demonstrated to occur by an increase in the electrostatics interactions and the transmembrane potential (Lemeshko, 2013). In this same study, the designed polycationic peptides revealed a selective anticancer activity against cancer human acute T cell leukemia which the authors attribute to higher values of surface and membrane potential of tumor cells when compared with normal cells (Lemeshko, 2013). Other different examples regarding the importance of electrostatics interactions in ACP—membrane interplay are patent on many studies such as the ones with the already described AMP polybia-MPI (Wang et al., 2009b) and with bovine lactoferricin 6 (LfcinB6) (Richardson et al., 2009). The short α-helical peptide polybia-MPI is selective toward leukemia cells probably due to the different amount of PS exposed in the cancer cell membrane (Wang et al., 2009b). Cell proliferation, viability and cytotoxicity assays revealed that polybia-MPI impaired the proliferation of sensitive and MDR cells while inducing LDH activity. On the contrary, the peptide showed lower effect on normal fibroblasts (Wang et al., 2009b). The mechanism of action relied on the disruption of the plasma membrane by pore formation which was shown by microscopy analyses. Upon contact with the negative cell membrane, the peptide acquires helical conformation capable of destroying the membrane (Wang et al., 2009b). Electrostatics is thus the main force attraction and the hydrophobic interactions allow the peptide insertion into the membrane. Leukemia cells died by a necrosis effect, with cells swelling and bursting (Wang et al., 2009b). Bovine lactoferricin (LfcinB) is a 25 amino acid residues cationic AMP isolated from cows' milk (Hoskin and Ramamoorthy, 2008). This peptide presents an amphipathic β-sheet configuration and displays anticancer activity against leukemia cells and various other carcinomas (Mader et al., 2005) being able to bind to GAGs of the cell membrane (Jenssen et al., 2004). LfcinB is capable of inducing apoptosis by direct disruption of the mitochondrial membrane (Furlong et al., 2008), but is also capable of lysing the membrane depending on the cancer cell type (Eliassen et al., 2006). LfcinB6 is the antimicrobial core of LfcinB peptide with a +3 net charge conferred by the three arginine residues in its sequence (Ueta et al., 2001). This charge is expected to promote bacteria death (Ueta et al., 2001) but it was insufficient for allowing the peptide binding and interaction with T cell leukemia membranes. Indeed, it was shown that a net charge of at least +7 was required for a strong cytotoxic activity toward the tumor cells (Richardson et al., 2009). On the other hand, the peptide showed cytotoxic activity when delivered by fusogenic liposomes into the cytosolic compartment of the same cells involving cathepsin B and caspase activities (Richardson et al., 2009). On a different study, CB1a, a cecropin-derived peptide, showed high cytotoxic activity against leukemia and stomach carcinoma with low hemolysis (Wu et al., 2009). In this case, the net positive charge of the peptide (+12) proved to be important for its activity even though the exact mode of action for this peptide is still poorly understood (Wu et al., 2009).

Even though peptides' net positive charge is shown essentially to have a promoting role in ACPs binding to the membrane negative charge of cancer cells, the negative components of the membrane might function also as inhibitors of the activity. Indeed, LfcinB and KW5, a derived peptide from LfcinB with 21 amino acid residues designed to adopt an idealized amphipathic α-helical structure, were shown to have decreased effect on lymphoma cells expressing heparan sulfate (HS) on the cell surface (Fadnes et al., 2009). It was proposed that the HS at the surface of cells sequester the peptide molecules leading them away from the membrane bilayer and thus poorly differentiated tumors with low expression of cell surface HS are more susceptible to peptides' activity (Fadnes et al., 2009). Apart from this inhibitory effect of the negatively charged molecules expressed on cancer cell surface, it is also possible to find peptides that seem insensitive to differences in the membrane charge, like SK84 (Lu and Chen, 2010). SK84 is a glycine-rich non-cationic AMP isolated from *Drosophila virilis* which membrane disruption activity of leukemia cells was evidenced by SEM (Lu and Chen, 2010). This disruption might be consequence of the perturbation of the membrane not due to electrostatic forces but by the formation of an elastic structure via the peptide flexible N-terminal glycine-rich domains (Lu and Chen, 2010). The SK84 peptide, with this unusual mode of action, is toxic to leukemia, liver and breast cancer cells, while remains non-toxic to mouse and human erythrocytes (Lu and Chen, 2010).

#### Peptide activity on non-solid tumors

Magainins are naturally occurring peptides isolated from the skin of *Xenopus laevis* that present antibiotic activity toward different microorganisms (Cruciani et al., 1991). These molecules are α-helical peptides with separate cationic and hydrophobic faces comprising 21–27 amino acid residues (Hoskin and Ramamoorthy, 2008). Cruciani et al. showed that nine synthetic magainin peptide analogs lyse in a rapid and irreversible way several hematopoietic tumors with a cytotoxicity comparable to their antibacterial activity and with relatively non-toxicity to differentiated normal cells, peripheral blood lymphocytes (PBLs), and polymorphonuclear lymphocytes (PMNs) (Cruciani et al., 1991). The study revealed that all the tested hematopoietic cell lines were sensible to the peptides derivatives with varying degrees of cytolytic activity within minutes. It also showed that the magainin derivatives were selective toward the tumor cells at concentrations 5–10 times greater than those required for antibiotic effects but 10–20 times less than those which are toxic to normally differentiated cells (Cruciani et al., 1991). In agreement to magainins described mode of action, the permeability of tumor cell membranes was affected by α-helical channel formation on the cell membrane while non-cytolytic concentrations of these peptides were not sufficient to form selective α-helical ion channels capable of compromising cell viability (Cruciani et al., 1991).

Also α-helical peptides, cecropin derivatives have been studied as potential alternatives for targeting leukemia cells (Srisailam et al., 2000; Wu et al., 2009). In a study conducted using a custom AMP, cecropin B1 (CB1), researchers point out that parallel to the importance given to peptide structure, the orientation of the peptide after approaching the surface of the polar lipid heads conditions peptides' activity (Srisailam et al., 2000). CB1 activity was compared with two amphipatic α-helical segments derived from the natural cecropin B (CB) and no selective activity for CB1 was described while the differences reported for the IC_50_ values for different leukemia cell lines have been attributed to the heterogeneity regarding the different tested cells. Thus, adding to the structure and helix stability and to peptide self-orientation, the peptide flexibility appears to be a key factor for the efficient insertion on the membrane in the first events of membrane lysis (Srisailam et al., 2000).

Apart from LfcinB, other peptides have been recently described as apoptosis inducers in leukemia cells (Edison et al., 2012; Labelle et al., 2012). Pep2 and Pep3 are short synthetic peptides derived from the C-terminus of the proapoptotic mitochondrial protein ARTS and were shown to efficiently kill cells from human leukemia (Edison et al., 2012). Also, a stapled peptide combining the Bcl-2 interacting mediator of cell death (BIM) with stabilized α-helix of Bcl-2 domain (SAHBs) named BIM SAHB_A_ was recently developed for targeting the Bcl-2 pathway (Labelle et al., 2012). This peptide disables survival proteins and activates the Bcl-2 family proteins resulting in cancer cell death by overcoming the apoptotic resistance expressed in hematological cancers (Labelle et al., 2012). Experiments showed that the peptide was able to suppress the growth of drug-resistant leukemia tumors in mice and also showed a synergistic anticancer effect when administered with other drugs (Labelle et al., 2012).

## Perspectives and open questions on anticancer peptides design and development

The use of peptides in clinical treatments has many advantages as well as drawbacks. The challenge in ACP designing lies on the improvements of their delivery to the tumors while maintaining a low profile of toxic effects. The low selectivity of some of the ACPs molecules, the high cost of production in large scale, and their low resistance to proteolytic cleavage (Hu et al., 2011) are some of the main reasons why peptides have been retained in drug development pipelines. There are also some concerns related to the use of AMPs whose sequences are close to human and natural AMPs due to a possible compromise of the human natural defense and consequently threat to public health (Chen et al., 2012). On a positive view, since ACPs are not directed to a specific extracellular or intracellular receptor, some mechanisms of resistance can be impaired (Giuliani et al., 2007; Torfoss et al., 2012b) and actually some AMPs have shown cytotoxic activity against MDR cancer cells (Johnstone et al., 2000). The success for obtaining an optimal ACP relies then on the manipulation of its sequence, net charge, secondary structure, oligomerization ability, amphipathicity and hydrophobicity while maintaining high serum stability. The result should be a balanced equilibrium between these characteristics. Although there are no defined rules for designing ACPs, some facts established through structure-activity studies might help in elucidating the lack of selectivity for some peptides and potentiate drug development strategies. During the rational drug designing process differences in the pattern expression of surface molecules or in membrane fluidity between malignant cells types which may dictate the preference of the peptides for certain cancer cells in detriment of others (Fadnes et al., 2011) should not be neglected. Peptides' ability in crossing the cell membrane to reach intracellular targets is a major requirement for developing an anticancer agent. Peptides' structure might condition its internalization as well as contribute exponentially to the productions cost. Identifying the amino acid sequence in the peptides full sequence which might be responsible for the anticancer activity will certainly help reducing the high cost production by allowing the synthesis of shorter fragments that retain full biological activity. Many pharmacological parameters will be improved with this process, such as bioavailability and stability, and also immunogenicity is expected to decrease (Fadnes et al., 2011). It might also be expected that the shorter peptides are more efficient in reaching the membrane phospholipid bilayer with a concomitant increase of peptides' cytotoxicity (Fadnes et al., 2011). One clear and recent example reports to FK-16 peptide, a fragment of LL-37 which is the only peptide from the cathelicidin familiy found in humans (Ren et al., 2013). This shorter fragment showed an improved anticancer activity when compared to the original sequence and was described as capable to kill colon cancer cells by autophagic cell death, an additional cell death pathway, while reducing cost production (Ren et al., 2013). It has been shown that arginine residues in cationic AMPs interact strongly with zwitterionic phospholipids which may result in toxicity events (Yang et al., 2003; Giuliani et al., 2007). It is therefore expected that other cationic residues might be used when constructing an ACP, such as lysine, to direct the peptide binding toward the negatively charged cells and simultaneously avoid hemolytic events. Serum stability might be improved by the incorporation of D-amino acids on the peptide sequence (Riedl et al., 2011b) and by cyclization of the structures (Torfoss et al., 2012b). The incorporation of lipophilic β^2, 2^ amino acids building blocks into heptapeptides resulted in a potent anticancer activity toward human and murine lymphoma cells, as well as high proteolytic stability and low toxicity against non-tumor cells (Torfoss et al., 2012a,b). The authors of this study state that the incorporation of the disubstituted β^2, 2^ amino acids in an α-helical peptide added an extra methylene group to the structure which in combination with two bulky lipophilic substituents, increased stability to protein degradation. Indeed, the central β^2, 2^ amino acid is flanked by two tryptophan residues to increase bulkiness and forming a lipophilic sequence where lysine cationic residues, are located at the N- and C-terminals (Torfoss et al., 2012a). The cyclization of these peptides resulted in structures with increased rigidity, amphipathicity and with changes in the secondary structure conformation which lead to an improvement of the anticancer potency against human lymphoma cells (Torfoss et al., 2012b).

The hydrophobicity of the peptides is also an important property when considered the hydrophobic environment that characterizes the cell membrane and it can be easily modulated in order to increase anticancer activity (Huang et al., 2011). In one recent study, the authors proved that manipulating the hydrophobicity of a 26-amino acid residues amphipathic peptide, V13K, by changing between alanine—leucine residues, it was possible to increase peptide activity against cancer cells, having human cervix cells shown high sensitivity, and thus showed a correlation between hydrophobicity—anticancer activity (Huang et al., 2011). Increasing hydrophobicity on the nonpolar face of the peptides enhanced their helical structure which in combination with the hydrophobicity resulted in stronger self-association and anticancer activity (Huang et al., 2011). In turn, helical structure acquisition can be controlled by D-amino acid substitution which may also modulate peptide specificity (Huang et al., 2012). The same study allowed the simultaneous observation of an increased hemolytic activity with the increase of the hydrophobicity of the peptides revealing a low degree of specificity. In 2013, a different study supported the same correlation between increasing hydrophobicity and anticancer and hemolytic activities (Yang et al., 2013). The authors suggest that high cationicity for enhancing neoplastic cell specificity and controlled hydrophobicity for equilibrating the hemolysis effect might be a suitable strategy for drug design (Yang et al., 2013).

Tumorigenesis is a multistep process where many factors intervene for the tumor growth and progression as well as in the metastatic and angiogenic events. For an effective targeting of each step, new therapeutic agents with the ability to kill slow-growing and drug resistant cancer cells, despite their proliferative capacity, are needed. The design of an oncolytic peptide with optimized properties and with high impact on the area of cancer treatment requires obtaining precise information concerning peptides' activity on the cell membranes at high resolution and detail. Once this goal is achieved, and combined with the advance knowledge on cancer biology, the optimized peptide may prove to be economically and therapeutically viable and a valuable alternative to current chemotherapeutics. These new chemotherapeutic drugs may synergize with the existing agents to restrict tumor activity and it is unlikely that they elicit multidrug resistance mechanisms and increase side-effects on healthy tissues and organs.

### Conflict of interest statement

The authors declare that the research was conducted in the absence of any commercial or financial relationships that could be construed as a potential conflict of interest.
